# Early Lessons From Working With Local Partners to Expand Private-Sector Health Care Networks in Burundi and Mali

**DOI:** 10.9745/GHSP-D-24-00109

**Published:** 2024-10-29

**Authors:** Lydia Gahimbare, Nina Shalita, Erin Files Dumas, Mariela Rodríguez, Pierre Moon

**Affiliations:** a Population Services International/Burundi, Bujumbura, Burundi.; b Population Services International/Mali, Bamako, Mali.; c Population Services International, Washington, DC, USA.

## Abstract

The private health care sector is an important source of service delivery in low- and middle-income countries (LMICs). Yet, the private sector remains fragmented, making it difficult for health system actors to support and ensure the availability of quality health care services. In global health programs, social franchising is one model used to engage and organize the private health care sector. Two social franchise networks, ProFam in West Africa and Tunza in East and Central Africa, provide health care through branded networks of facilities. However, these social franchise networks include a limited number of private health care facilities, and in fragile contexts, like Burundi and Mali, they have faced challenges in integrating with national health systems. The MOMENTUM Private Healthcare Delivery (MPHD) project in Burundi and Mali sought to expand the number of health facilities it engaged beyond the existing ProFam and Tunza networks. The expansion aimed to help improve service quality in more private facilities while advancing localization and reducing fragmentation for improved stewardship by health system actors. MPHD achieved this expansion by removing barriers for private health facilities to join inclusive, nonbranded networks and engaging local partners to build and maintain these networks. We share lessons learned regarding the growing role of local organizations as actors within mixed health systems and provide insights on strengthening stewardship of the increasingly heterogeneous private health care delivery sector in LMICs, particularly in fragile settings.

## Abstract (precis)

Expanding private health care delivery networks is possible through partnerships with local organizations. This article explores how expanded networks differ from traditional social franchise networks and discusses how they can support and steward private health care facilities in contexts like Burundi and Mali.

## Teaser

**Key Findings:**

- Working with local partners supports the expansion of private health care networks in a form that differs from traditional social franchises and that enables support to and stewardship of a larger number of private health care facilities.
- Expanded private health care networks include a diverse array of providers and facilities and require flexibility in defining criteria for inclusion, supporting quality of care, and collecting and reporting monitoring data.
- Some local partners are well positioned to support private health care facilities and represent the private health care sector in national and subnational fora. However, they require support and capacity-strengthening to realize and sustain these roles.

**Key Implications:**

- One approach to organize the private health care sector and reduce fragmentation is through expanding networks of health facilities, which may be particularly important in low-income and fragile contexts where there is limited stewardship of the private health care sector.
- Donors and international nongovernmental organizations should continue to invest in and support the capacity-strengthening of local partners if they are to play meaningful roles in improving private health care delivery.

## BACKGROUND

The private sector plays an important role in health care in many countries, including in functions such as new technology development, supply chain management, and the training of human resources for health.^1^ Regarding service delivery, according to the World Health Organization, the private sector in Central, East, Southern, and West Africa provides an estimated 35% of all outpatient care^1^ and serves various wealth quintiles.^2^ However, the providers and facilities that make up the private sector are increasingly diverse^3^ and remain fragmented,^2^ particularly in low- and middle-income countries (LMICs). This fragmentation hampers critical health system functions, such as workforce training, quality assurance, data sharing, supplies provision, financing, and community engagement.^4^ Platforms and federations to organize and mobilize the private health care sector at regional, national, and subnational levels exist in different forms,^5^ including across different regions in Africa.^6^ These entities demonstrate that private health care sector actors themselves see a benefit to aggregating with others and reducing fragmentation.

At the service delivery level, social franchising is one of several models used to organize and engage the private health care sector, especially for the delivery of reproductive health services, such as voluntary family planning (FP).^7^^–^^9^ It does so through the creation and maintenance of networks of health facilities that are designed to increase the availability and quality of services. Social franchising can take various forms. Full franchising sees the franchisor focus on the entirety of the health care practice, whereas the more common fractional franchising approach sees the franchisor focus on specific clinical areas.^4^ Fractional franchising has been most documented in the literature, and some programs have demonstrated increased client volume and accessibility in health care facilities.^7^^,^^10^ However, social franchising has faced challenges regarding cost-effectiveness and sustainability.^4^^,^^11^ Further, social franchise networks are exclusive by design: franchisees are selectively recruited^4^ and must meet specific criteria to join and remain within the network.

ProFam (in West Africa) and Tunza (in East and Central Africa) are 2 social franchises led by Population Services International (PSI) that provide health care through branded networks of facilities. Historically, they have focused primarily on FP services, with other health areas included, depending on the country and context.^4^ Although they use a common brand across these regions, ProFam’s and Tunza’s scale and scope differ according to the context of each country. In least developed countries^12^ and particularly in fragile contexts like Burundi and Mali,^13^ the integration of social franchise facilities into national systems has proved challenging and has been limited by the public sector’s capacity to carry out the necessary stewardship functions^14^ in the private sector. International nongovernmental organizations (INGOs) and donors continue to play key stewardship roles in these contexts. However, PSI has also experienced the emergence of diverse local partners (including national nonprofit and faith-based organizations, youth networks, professional associations, and private-sector platforms) working with the private health care sector in various capacities, providing an opportunity to expand stewardship of private health care facilities and, ultimately, expand the availability of quality health services.

Estimates of the private health care sector in Burundi and Mali identified 525 and 717 primary-level private health facilities present in Burundi and Mali, respectively.^15^^,^^16^ Approximately 16% and 20% of FP users in Burundi and Mali, respectively, obtain their contraceptive methods in the private sector.^17^^,^^18^ In both countries, there is limited public-sector stewardship of and support for private health care service delivery. Training, supervision, and data collection in the private sector may be sporadic or missing.

To better organize small- to medium-sized private health care facilities within the wider health system and support these facilities to deliver quality services, the MOMENTUM Private Healthcare Delivery (MPHD) project was launched in Burundi and Mali in 2021 (Table). The project is funded by the U.S. Agency for International Development (USAID) as part of the MOMENTUM suite of awards and implemented by PSI with partners Jhpiego, FHI 360, Avenir Health, and ThinkWell, as well as local partners across several countries, including those discussed in this article.

The project initiated a novel approach to building and maintaining health facility networks, expanding beyond Tunza (in Burundi) and ProFam (in Mali). The novel approach included removing barriers for private health facilities to join inclusive, nonbranded networks and engaging diverse local partners to build and maintain these networks.

The novel approach to building and maintaining health facility networks included removing barriers for private health facilities to join inclusive, nonbranded networks and engaging diverse local partners to build and maintain these networks.

In contrast to full or fractional franchising, this novel approach emphasized the inclusion of private, nonbranded health facilities that offered a range of services. Expanding beyond original social franchise networks meant reaching a wider subset of the private sector and helping to address the limited stewardship and support experienced by the private health care facilities in the 2 countries. The approach was also influenced by the increasing focus on localization in the global health community,^19^^–^^21^ which recognizes local leadership and ownership as essential to sustainable development^22^ and saw local partners either oversee a certain number of facilities in the network (Burundi) or certain functions within the health facilities in the network (Mali).

In this article, we provide insights on strengthening stewardship of the increasingly heterogeneous private health care delivery sector in LMICs, particularly in fragile settings like Burundi and Mali, and share lessons learned regarding the growing role of local organizations as actors within mixed health systems.

## APPROACH

### Identifying Private Health Facilities

To increase the number of private health care facilities with which the project engaged, MPHD deployed an inclusive recruitment strategy for private health facilities to join expanded networks. This included consulting national facility registers, relevant authorities, and various local organizations and entities to develop lists of private facilities operating in the countries. Health facility assessments were conducted to determine the scope, level, and quality of health care services provided.

### Removing Barriers to Joining Facility Networks

Similar to social franchising, participation of the health facilities in the expanded networks was voluntary and dependent upon the owners’ and providers’ interest. Basic requirements included legal status to operate and willingness to share service delivery data. However, in contrast to social franchising, health facilities in the expanded networks were not required to offer a specific set of clinical services or have a certain type or number of providers. Health facilities were supported to define their own success, be that in expanding service offerings, increasing client volume, or retaining their existing human resources. These objectives were not defined by the wider network. Further, because there was no logo or name attached to the network, they were neither incentivized nor required to adhere to a social franchise brand. Facilities that were affiliated with the ProFam or Tunza brands could maintain that branding and affiliation. Health facilities were motivated to join the expanded networks to access resources, such as provider trainings, demand creation channels, and support to improve health facility management (e.g., data, inventory, and financial management).

Taking this approach, the expanded network included more sites compared to the ProFam and Tunza networks and covered a greater proportion of the primary-level private health facilities in each country (Figure).^15^^,^^16^ This approach reached 1,036 providers in Burundi and 855 providers in Mali with training and supervision on clinical quality and facility management.

This inclusive approach reached 1,036 providers in Burundi and 855 providers in Mali with training and supervision on clinical quality and facility management.

### Engaging Local Partners to Build and Maintain Networks

Health facility networks are typically facilitated by third-party aggregators. PSI and other INGOs have historically played this role in social franchise networks,^9^ providing specific inputs, such as trainings, supervision, commodities access, and oversight of data collection at the supported health facilities. Under MPHD, local partners were identified and engaged to build and maintain these new, expanded networks.

MPHD conducted a mapping of organizations with existing ties to the private health care sector in each country. In Burundi, this exercise identified the Association Nationale pour le Franchise Sociale (National Association for Social Franchise, ANFS), which engaged with Tunza-branded private health facilities, and the Réseau des Confessions religieuses pour la promotion de la santé et le Bien-être Intégral de la Famille (Network of Religious Confessions for the Promotion of Health and Integral Well-Being of the Family, RCBIF), which engaged private faith-based health facilities. In Mali, MPHD identified the L’Alliance du Secteur Privé pour la Promotion de la Santé au Mali (Private Sector Alliance for the Promotion of Health in Mali, ASP-PSM), which operates at the national level as a representative body of the private health sector. Other organizations identified in Mali included JIGI, a local nongovernmental organization focused on social marketing; the Association des Sages-Femmes du Mali (Association of Midwives of Mali, ASFM), which spans both the public and private sectors; and the Réseau des Jeunes Ambassadeurs pour la SR/PF au Mali Network of Youth Ambassadors for FP/RH in Mali, RJAM), which focuses on demand generation.

Once engaged with the MPHD project as local partners, these organizations leveraged existing ties with private health facilities to bring those facilities into the expanded network. For example, RCBIF identified faith-based health facilities of various denominations with which it had worked in the past. ASP-PSM, alternatively, leveraged ties with private-sector facilities it had previously supported under other donor funding.

Following selection and onboarding of these sites into the expanded networks, MPHD worked to strengthen local partners’ capacity in core functions of network management and direct support to health facilities. Capacity-strengthening activities were tailored to the different roles of each organization. For example, local partners with clinical staff were trained in how to conduct clinical training and clinical supportive supervision. Those with advocacy objectives related to private-public engagement were trained in data aggregation and visualization. Those supporting commodities procurement were supported in stock management and quantification. Coordination between these partners was facilitated by the MPHD project and, in certain circumstances, through integration with the wider health system. For example, in Mali, MPHD fostered coordination between ASFM, which was conducting supportive supervision, and ASP-PSM, which was supporting the use of electronic medical records and data management systems. Alternatively, in Burundi, both ANFS and RCBIF commenced participation in fora hosted by the national-level public health authorities, meaning coordination between these partners also took place in conjunction with the public sector.

With the project’s support and facilitation, all local partners also developed organizational capacity-strengthening plans and have been conducting activities to enhance their institutional development and business skills to improve securing and managing donor funding. Given the political and economic circumstances in Burundi and Mali, it is expected that ongoing support to the expanded health care networks will need to be sustained through external donor funding in the short term, and local partners will require necessary skills to manage those funds. However, the working assumption of MPHD has been that strengthened local capacities, deepened interrelationships among local actors, and the devolution of more leadership and ownership to local actors creates possibilities for stronger health markets in the long term.

Presently, each of the local partners is conducting specific activities to support the maintenance of the expanded networks through the MPHD project. In Burundi, ANFS and RCBIF are each responsible for supporting key functions of a subset of the facilities within the wider network. In Mali, these functions are divided up across the local partners, with ASFM supporting clinical functions, RJAM supporting demand generation, JIGI supporting product access, and ASP-PSM supporting governance and representation within the wider health system.

## LESSONS LEARNED

The MPHD project seeks to better organize large numbers of private health care facilities and support these facilities to deliver quality services. In the fragile contexts of Burundi and Mali, where public-sector stewardship of the private sector faces limitations, the project sought to elevate the role of diverse local organizations to perform stewardship functions, such as enhancing health data collection within private facilities, facilitating support to and oversight of health care workers, and strengthening private and public coordination and dialogue. Where applicable, the project facilitated coordination of these efforts with those of national health authorities and, in all cases, supported local organizations to build their institutional capacity to leverage current and future resources to improve private health care coordination and delivery.

We share the following insights and lessons learned from expanding health facility networks and engaging diverse local partners to help build and maintain these networks.

### Building and Maintaining Expanded Networks Is Possible

It is possible and effective to build and maintain expanded networks of private health facilities that differ from traditional social franchises. Engaging with a larger number of health facilities has brought more coherence and visibility to the private health care sector in places where there was limited stewardship thereof. In Burundi, all sites in the expanded network are now routinely reporting monitoring data into the national health information system, and these data are verified through periodic quality audits conducted jointly by the project and the Ministry of Public Health. This level of data visibility enables more targeted provider capacity-strengthening at specific sites with high volumes of services, promotes data for decision-making, and can help optimize how initiatives like MPHD can best allocate their training resources.

Engaging with a larger number of health facilities has brought more coherence and visibility to the private health care sector in places where there was limited stewardship.

### Private Health Facility Networks Require Flexibility to Accommodate Diversity

Because private health facilities differ greatly in the services they offer, the clinicians they staff, and their physical infrastructure, the network must be flexible and adaptable to accommodate this diversity if larger numbers of facilities are to be brought into the networks. In both countries, in addition to focusing on service quality, facility owners and providers identified their own goals for their health facilities. In some cases, the facilities sought to expand or diversify their clinical offerings, such as by increasing laboratory capacity, expanding maternity wards, or expanding the range of products or medications sold at their in-clinic pharmacies. Others were specifically interested in how to improve their stock management and forecasting. Working with the varied health facilities to understand their motivations to be a part of a network was an integral part of the health facility assessments and aligns with the principles of private-sector engagement.^23^ Further, this diversity has shown some promise for peer learning and experience-sharing, particularly when private health care owners and providers have convened under the auspices of the MPHD project or alongside the local partners at meetings convened by public health authorities.

### Tools to Support Quality Service Delivery Must Accommodate Diverse Networks

The scale and diversity of sites under the expanded networks present challenges on how to support quality of care. MPHD has addressed this challenge by supporting project staff and local partners to conduct needs-based quality reviews using the digital, open-source Health Network Quality Improvement System (HNQIS),^24^ which can function on any Android operating system, including smartphones, and operates through DHIS2, a data platform used in many LMICs. HNQIS encompasses a wide range of quality checklists for different services, of which those supporting and supervising the health facilities can select from a menu. HNQIS also helps supervisors (including those coming from local partners) identify providers and sites with the greatest gaps in quality, so visits to these providers can be prioritized. In Burundi, the 2 local partners use HNQIS, and, on average, quality scores have increased across sites. The quality scores for FP averaged 77 of 100 points in January–March 2022 and improved over time to reach 90 of 100 points in April–June 2023. Because HNQIS operates through DHIS2, quality scores and data analytics can be shared with the wider health system. Adaptive tools and solutions like HNQIS will be essential to support any broad and diverse network of private health facilities.

### Local Partners Support Links Between the Public and Private Health Care Sectors

In addition to supporting the health facilities directly, local partners have leveraged their roles as network facilitators to strengthen stewardship of data, commodities, and health worker capacity-strengthening, as well as supporting certain health facilities to explore enrollment in national insurance and/or financing mechanisms. In Mali, ASP-PSM is orienting private health care facilities on updated national health policies and supporting efforts for interoperability of private facility electronic medical records with national health information systems. In Burundi, ANFS is now a part of periodic national data reviews that cover all public and private health facilities. This has normalized the inclusion of private-sector data across national efforts, including commodities quantification. These experiences demonstrate that local partners have the potential to create robust connections with the wider health system when they represent a wider number of health facilities than in the previous social franchise networks.

### This Model Provides an Opportunity to Strengthen Local Capacity

In working with local partners to conduct organizational capacity assessments and realize capacity-strengthening plans to improve internal processes and controls, MPHD has seen improvements in areas including governance, project management, and human resource management. However, limitations persist for local partners to operate under financial controls required by donors, reformulate or enhance organizational processes (e.g., procurement), and define and maintain indirect or overhead rates. As local partners move into network facilitation roles, organizational capacity-strengthening is needed, especially in the areas of finance and compliance, to grow and sustain these roles.

## CONCLUSION

Health systems in contexts such as Burundi and Mali are likely to continue to face challenges in stewarding and supporting small- to medium-sized private health facilities. Working with local partners to organize networks of private health facilities is one way to support a more cohesive, mixed health system. This approach aligns with overall principles of organizing the private sector to facilitate market development, which is also undertaken by strengthening other private health care federations and associations that operate at various levels across sub-Saharan Africa. Such investments benefit facilities and providers by having access to training and support for improved quality, as well as the health system by having comprehensive data from and stewardship of the private sector. As local partners continue to expand and develop their roles as network facilitators, external funding and support will be needed to sustain these efforts. With continued focus on localization, INGOs that have historically worked as social franchise aggregators may be well positioned to support local partners to facilitate expanded networks of health facilities.

**FIGURE:**
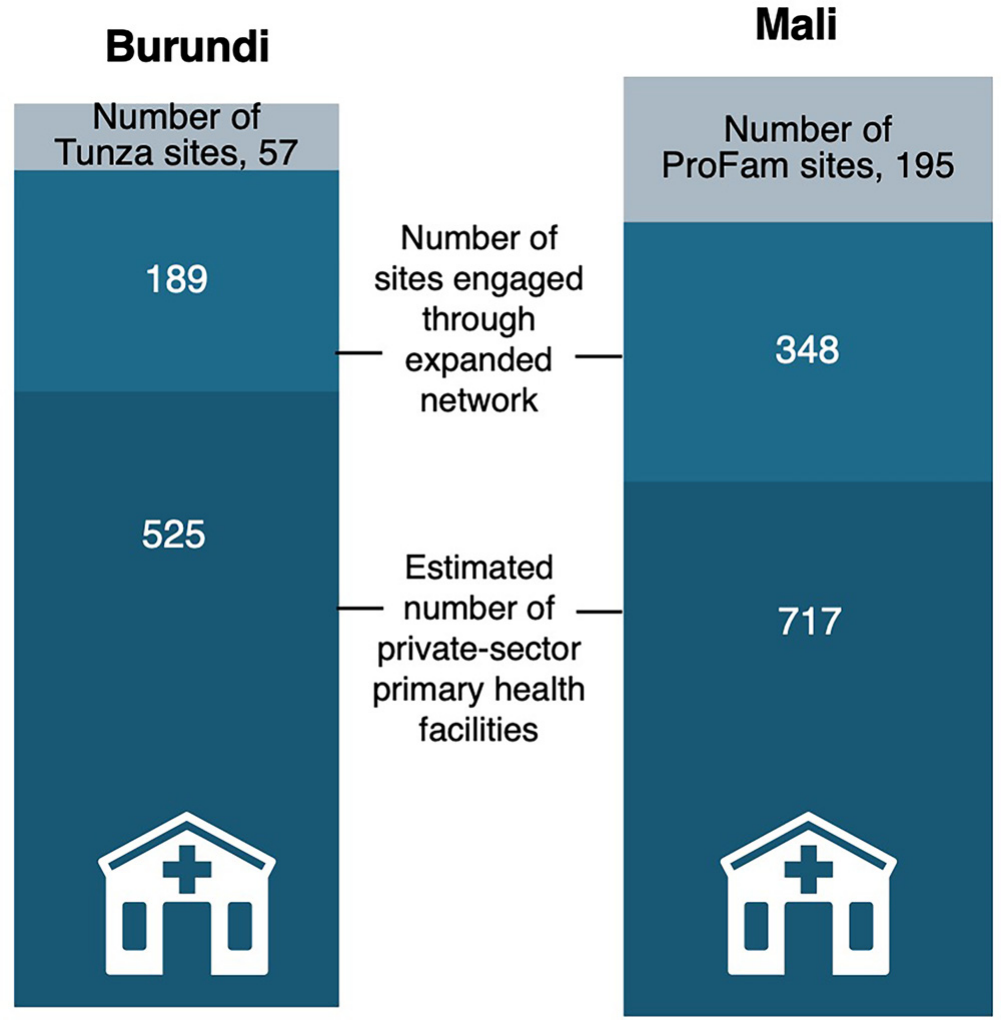
The Reach of the Social Franchise and Expanded Networks Relative to the Total Estimated Number of Private-Sector Primary Health Facilities in Burundi and Mali

**TABLE.** MOMENTUM Private Healthcare Delivery Project, Burundi and Mali, 2021–2024

	**Burundi**	**Mali**
Small- to medium-sized health care facilities supported, no.	189	348
Geographic coverage	18 of 18 provinces	5 of 10 regions, and the Bamako Capital District. The majority of Mali’s population lives in these areas.
Types of private facilities supported	Tunza franchised, independent, faith based	ProFam franchised, independent, community run
Local partners	Association Nationale pour le Franchise Sociale Réseau des Confessions religieuses pour la promotion de la santé et le Bien-être Intégral de la Famille	L’Alliance du Secteur Privé pour la Promotion de la Santé au Mali Association des Sages-Femmes du Mali JIGI Réseau des Jeunes Ambassadeurs pour la SR/FP au Mali
Health services supported	Family planning/reproductive health Maternal and newborn health Treatment of childhood illnesses Malaria prevention, diagnosis, and treatment	Family planning/reproductive health Maternal and newborn health Treatment of childhood illnesses Nutrition Water, sanitation, and hygiene COVID-19

## Notes

**Peer Reviewed**

**First Published Online:** October 11, 2024.
